# Clinician Perceptions of Barriers and Strategies to Improve Pediatric Hypertension Detection

**DOI:** 10.1001/jamanetworkopen.2025.60542

**Published:** 2026-02-27

**Authors:** Abbas H. Zaidi, Erica Sood, Sarah De Ferranti, Fabliha Khurshan, Samuel Gidding, Varsha Zadokar, Jonathan Miller, Anne Kazak

**Affiliations:** 1 Nemours Cardiac Center, Nemours Children’s Health, Wilmington, Delaware; 2 Department of Pediatrics, Sidney Kimmel Medical College, Thomas Jefferson *University, Philadelphia, Pennsylvania; 3 Center for Healthcare Delivery Science, Nemours Children’s Health, Wilmington, Delaware; 4 Division of Ambulatory Cardiology, Department of Cardiology, Boston Children’s Hospital, Boston, Massachusetts; 5 Department of Pediatrics, Harvard Medical School, Boston, Massachusetts; 6 Department of Genomic Health, Geisinger, Danville, Pennsylvania; 7 Department of Pediatrics, Nemours Children’s Health, Wilmington, Delaware

## Abstract

**Importance:**

Pediatric hypertension remains underrecognized despite its high prevalence (approximately 5%) and long-term cardiovascular implications. Although guidelines for screening and diagnosis exist, less than 25% of affected children are diagnosed, and more than half do not receive timely follow-up care. Clinician-specific implementation barriers and directly informed strategies, which may alleviate this gap, are insufficiently described.

**Objective:**

To identify clinician-reported barriers and actionable recommendations to improve pediatric hypertension detection and follow-up in primary care using the Consolidated Framework for Implementation Research.

**Design, Setting, and Participants:**

Semistructured qualitative interviews were conducted between November 1, 2022, and March 31, 2023, with health care clinicians at 10 pediatric primary care clinics. Clinics were purposefully selected based on outpatient follow-up care gaps for patients 1 year or more after initial hypertension diagnosis and the Child Opportunity Index based on clinic address.

**Exposures:**

Transcripts were analyzed thematically and mapped to Consolidated Framework for Implementation Research domains and constructs to identify modifiable barriers and stakeholder-informed recommendations.

**Main Outcomes and Measures:**

Clinician-identified barriers and recommended strategies for improving pediatric hypertension detection and follow-up.

**Results:**

A total of 25 health care clinicians were interviewed (mean [range] age, 45.1 [28.0-64.0] years; 22 [88%] female). A total of 10 themes emerged; 5 major barriers to hypertension detection were identified that directly aligned with 5 themes for recommendations. Clinicians described (1) lack of standardized clinical pathways, with the recommendation to implement role-specific pathways for blood pressure measurement and follow-up; (2) insufficient training and confidence among clinicians and staff, addressed by competency-based and continuous training programs; (3) electronic medical record systems that did not support blood pressure trend analysis or decision-making, with the recommendation to redesign and optimize integration; (4) variable access to properly maintained and sized equipment, with the recommendation to ensure reliable availability and calibration; and (5) fragmented referral and communication with subspecialists, addressed by establishing structured, shared communication and referral systems.

**Conclusions and Relevance:**

This qualitative study demonstrated that improving pediatric hypertension detection requires targeted strategies that address clinician-level implementation barriers. Stakeholder-informed recommendations offer a roadmap to operationalize clinical guidelines that can improve hypertension detection.

## Key Points

**Questions:**

What are barriers to detection of hypertension among children in primary care settings, and what strategies do clinicians recommend to address these barriers?

**Findings:**

In this qualitative study of 25 clinicians across 10 pediatric primary care clinics, barriers included lack of standardized clinical pathways, inadequate training, inefficient electronic medical records, inconsistent equipment access, and poor coordination with specialists. Clinicians recommended standardized role-specific pathways, competency-based training, optimized electronic medical record tools, reliable equipment systems, and structured communication with subspecialists.

**Meaning:**

This study’s results suggest that addressing clinician-identified barriers through targeted, system-level strategies may improve pediatric hypertension detection and management.

## Teaser

This qualitative study of clinicians in pediatric primary care clinics explores clinician-identified barriers to hypertension detection and addresses these barriers in alignment with the Consolidated Framework for Implementation Research.

## Introduction

Pediatric hypertension affects approximately 3% to 5% of children with cardiovascular, kidney, and cerebrovascular injury, leading to long-term morbidity and mortality.^1,2,3,4,5,6,7,8^ Evidence of target organ damage from hypertension, including carotid intimal thickening and left ventricular hypertrophy, appears as early as childhood, serving as precursors to future disease.^1,2,3,4,5,6,7,8^ Guidelines from the American Academy of Pediatrics specify age- and height-based blood pressure (BP) percentiles and emphasize confirmation of diagnosis by using BP measurements at 3 separate visits.^1^ Unfortunately, implementation of these steps in clinical settings remains inconsistent.^1,9,10,11,12^ National estimates indicate that fewer than 1 in 4 children with hypertension are identified and more than 60% do not receive recommended follow-up.^9,10,13^

Although prior research has examined parent and clinician knowledge gaps and perceptions related to pediatric hypertension,^9,13,14^ few studies have systematically investigated barriers to implementation of guideline-directed hypertension detection from the perspectives of health care clinicians responsible for hypertension diagnosis. Pediatric hypertension detection is a multistep process requiring accurate measurement, clinical decision-making, and coordination across various roles in primary care.^1^ Breakdown at any point, whether due to training gaps, unclear workflows, electronic medical record (EMR) limitations, or care coordination failures, can lead to poor hypertension detection.

This study aimed to identify clinician-identified barriers to hypertension detection and their recommendations to address these barriers in alignment with the Consolidated Framework for Implementation Research (CFIR).^15^ Use of this implementation science–informed framework enabled a structured, theory-based organization of findings and supported the identification of practical, clinician-informed strategies to improve pediatric hypertension detection.

## Methods

### Study Design and Setting

This study is part of a larger qualitative study on pediatric hypertension detection conducted across 10 pediatric primary care clinics in Delaware and Pennsylvania affiliated with a large regional pediatric health system conducted between November 1, 2022, and March 31, 2023.^12,16^ Clinics were purposefully selected based on 2 criteria: (1) the percentage of patients who lacked clinic follow-up care for 1 year or more after initial hypertension diagnosis (ranging from 0.9% to 28%) and (2) the Child Opportunity Index based on clinic location address, which measures neighborhood resources that support child health and development.^17,18,19^ A detailed description of clinic selection methods can be found elsewhere.^12,16^ The study was approved by Nemours Children’s Health institutional review board. The analytic process followed best practices for qualitative research, including adherence to the Consolidated Criteria for Reporting Qualitative Research (COREQ).^20^

### Participants

We recruited 25 health care clinicians across the 10 pediatric primary care clinics, including physicians, nurses, nurse managers, and medical assistants. Purposive sampling was used to capture variation in clinical roles, years of experience, and clinical characteristics. All participants provided written informed consent.

### Data Collection

Semistructured interviews were conducted via telephone by a trained research coordinator (V.Z.). The interview guide was informed by CFIR.^15^ Interview questions were aligned to the main CFIR domains and constructs of interest as indicated in eTable 1 in Supplement 1. Interviews were audio-recorded using Microsoft Teams, version 1.0 (Microsoft Corp) and transcribed using the platform’s automated transcription function. Research coordinators subsequently reviewed each recording in full, edited transcripts for accuracy, and removed all identifying information before analysis. In addition, the principal investigator (A.H.Z.) reviewed data obtained throughout the study in weekly meetings with a research coordinator (V.Z.). Based on ongoing transcript review, thematic saturation was reached by the 18th interview. To confirm saturation, interviews were continued through a total of 25 clinician participants, at which point no new themes emerged.

### Statistical Analysis

Interview data were analyzed using an inductive thematic approach.^21^ Two team members, a pediatric cardiologist (A.H.Z.) and a psychologist (E.S.), conducted open coding, iteratively refining the codebook through review of initial transcripts and discussion. Four clinician interviews were independently coded by both team members, achieving high intercoder reliability (pooled Cohen κ = 0.91), after which the remaining transcripts were divided between the coders. As subsequent transcripts were analyzed, no substantive new codes emerged after the initial coding; therefore, earlier transcripts did not require recoding. CFIR was applied as an analytic organizing framework after inductive coding was completed.

Thematic analysis was conducted in 2 main steps. In the first step, excerpts coded as clinician-reported barriers and recommendations were analyzed to develop themes (eg, lack of standardized clinical pathways). Next, we mapped each theme into a relevant CFIR domain and construct, which helped illuminate the broader context that influences implementation (eg, the aforementioned theme example falls under intervention characteristics: design quality and packaging). This theory-informed organization enhanced consistency and supported interpretation of findings within an implementation context. In the second step, we compared barriers and recommendations within the same CFIR domain to see where they matched with each barrier subtheme aligning with actionable strategies under the recommendation theme. For instance, the barrier of lack of standardized clinical pathway aligned with the recommendation to standardized clinical pathway for BP measurement. We re-reviewed these pairs to ensure the recommendation directly addressed the barrier and was actionable. This dual-step process enabled us to generate specific implementation-relevant recommendations linked to modifiable barriers reported by clinicians. These interconnected and linked barrier-recommendation themes are reported in this article. Although all barriers were coded, this analysis focused on those that could be mapped to feasible, practice-based strategies. Broader structural challenges, such as limited staffing or time constraints, were acknowledged but not included in the barrier-recommendation pairing because they did not yield corresponding, actionable recommendations within the scope of this study. The team held regular analytical debriefings to review code applications and theme development. In addition, to validate findings through member-checking,^22^ a subset of clinicians, including primary care physicians (PCP) and subspecialists (weight management and nephrology), reviewed the preliminary themes from the clinician interviews and confirmed their accuracy and validity.

## Results

Twenty-five health care clinicians participated in interviews between November 1, 2022, and March 31, 2023; 22 (88%) were female, 3 (12%) were male, and the mean (range) age was 45.1 [28.0-64.0] years. The sample of clinicians were from a range of clinic locations based on Child Opportunity Index and gaps in hypertension care (eTable 2 in Supplement 1). Participants varied in years of clinical experience, education background, and practice environments (eTable 3 in Supplement 1).

Five major themes representing barriers to hypertension detection were identified from the qualitative data, each aligned with specific CFIR domains and constructs (eTable 4 in Supplement 1). In addition, 5 major themes reflecting multilevel implementation strategies based on clinician-generated recommendations for improving hypertension care also were identified, paired with the barrier themes, and mapped to the CFIR domains and constructs. Thematic saturation was achieved, and each theme was endorsed by most participants. Themes related to barriers and recommendations and their corresponding CFIR domains and constructs are summarized in Table 1. Recommendations and representative excerpts are given in Table 2.

**Table 1.** Clinician-Reported Barriers and Aligned Recommendations for Pediatric Hypertension Detection, Organized by CFIR Domains and Constructs

CFIR domain and construct	Barriers	Aligned recommendations
Intervention characteristics: design quality and packaging	Lack of standardization in hypertension guidelines	Standardized clinical pathways for BP measurement
Refers to how well an intervention (such as BP guidelines) is designed, presented, and packaged for clinical use. If materials are confusing, incomplete, or not user-friendly, implementation is challenging.	Practitioners struggle with adherence due to lack of awareness and perceived complexity Guidelines are inconsistently applied across clinics, leading to variability in care Limited awareness of post-BP follow-up recommendations result in inconsistent patient management	Develop role-specific guidelines to clarify steps for BP measurement and follow-up Improve accessibility of guidelines via smart phrases, printed workflow documents, and quick-reference tools Establish clinical decision-making pathways for BP re-measurement and referral
Characteristics of individuals: knowledge and beliefs	Limited training and knowledge gaps	Competency-based training and educational resources
Captures the training, knowledge, and confidence of the people expected to use the intervention (eg, pediatricians, nurses, and MAs). If they are unaware of the guidelines, or do not believe they can apply them, implementation becomes difficult.	Practitioners lack adequate training on BP measurement, particularly in younger children Uncertainty in guideline application (eg, when to repeat BP readings and manual vs automated BP methods) Some practitioners lack confidence in initiating hypertension treatment Limited access to educational resources for both practitioners and caregivers	Implement regular role-specific training for MAs, nurses, and practitioners Conduct competency assessments on BP measurement, cuff selection, and workflow adherence Provide quick-reference materials in all examination rooms (eg, visual aids and laminated guides) Provide regular and ongoing training in BP measurements
Inner setting: compatibility and available resources (EMR challenges)	Inefficiencies in EMR for BP management	Optimized EMR integration for hypertension detection
Assesses whether the intervention fits with the organization’s existing systems, such as the EMR system. If the EMR does not flag abnormal BPs clearly or creates alert fatigue, it blocks effective implementation.	EPIC (health record software) inconsistently flags BP readings (missed, persistent, or unclear thresholds) No easy way to track BP trends during multiple visits Alert fatigue leads to practitioners ignoring or skipping notifications No hard stops preventing EMR closure before addressing BP abnormalities	Enhance EPIC’s BP tracking system with automated percentile data and trend visualization Implement hard stops requiring BP documentation before EMR closure Improve alert sensitivity to reduce excessive flagging and prevent practitioner desensitization Automatically pull BP percentiles into the notes
Inner setting: available resources (equipment and infrastructure)	Inadequate BP equipment and accessibility issues	Ensuring readily available and well-maintained BP equipment
Focuses on whether the right tools (such as BP cuffs, calibrated machines, and accessible storage) are available and reliable. Without these, clinicians cannot follow through even if they know what to do.	Limited access to properly sized BP cuffs, especially for infants and adolescents Uncalibrated or malfunctioning BP machines contribute to measurement errors BP machines and cuffs are not always stored in accessible locations, causing delays in patient care	Standardize equipment availability across all clinics to ensure access to properly sized cuffs and machines Implement routine calibration protocols to maintain measurement accuracy Organize storage systems to facilitate quick and easy access to BP tools
Process: engaging (coordination with subspecialists)	Limited PCP-specialist coordination in hypertension care	Strengthening PCP-specialist communication and referral systems
Examines how well key people outside the immediate team (such as subspecialists) are involved. Poor coordination creates gaps in referral and follow-up.	Lack of clear referral criteria for cardiologists and nephrologists PCPs have limited direct communication with specialists, delaying decision-making School-based health centers are underused for BP rechecks	Develop clear referral guidelines to ensure timely specialist consultation Enhance EPIC messaging to facilitate direct practitioner-to-specialist communication Use school-based clinics for BP monitoring and follow-up

Abbreviations: BP, blood pressure; CFIR, Consolidated Framework for Implementation Research; EMR, electronic medical record; MA, medical assistant; PCP, primary care practitioner.

**Table 2.** Aligned Recommendations, Actionable Steps, and Illustrative Excerpts From Health Care Practitioners to Improve Pediatric BP Measurement and Follow-Up

Aligned recommendations (major themes and actionable steps)	Excerpts
**Standardized clinical pathways for BP measurement (80%)**	
Develop role-specific guidelines to clarify steps for BP measurement and follow-up Improve accessibility of guidelines via smart phrases, printed workflow documents, and quick-reference tools Establish clinical decision-making pathways for BP remeasurement and referral	“I think it would be helpful to have a clinical pathway with what to do if there is a high blood pressure...as a standard guideline for providers...I don’t know if there’s like a specific smart phrase or dot phrase that would be utilized to be entered into the charts that would notify provider upon opening it that the patient had a high blood pressure other than them reviewing the vital signs.” HCP 11 “I don’t think we really have too much of a problem with measuring blood pressure. I think the next steps after that is what primary care pediatricians could really use help with...so I think this is a beautiful opportunity for a clinical pathway; sort of decision tree support and what to do next and what maybe what studies or interventions we could do to prevent unnecessary referrals...when to refer... just decision support on what to do after we make that diagnosis and even how to officially make the diagnosis. I think for the MAs, their job is really a technician type job, so they need to just be able to make the measurement of the blood pressure... Same for the nurses really. Every office has their nurses functioning in a little bit different way... So I think maybe some guidance for them in terms of follow up and having walking patients through our families through getting those additional blood pressures and time intervals of when those should be done that could be helpful for everybody. And then certainly for our providers though, what to do with the information we have is really would be very helpful.” HCP 3 “Like I have to say, like more illustration. Diagrams to like having a steps what to do when the patient is having high blood pressure when measured, so like the steps to everybody being on the same page and follow same protocol. That way everybody knows what to do and in those in those scenarios.” HCP 13 “I guess maybe a clinical pathway that we could follow a little more specifically than having to look up the AAP guidelines.” HCP 14
**Competency-based training and educational resources (92%)**	
Implement regular role-specific training for MAs, nurses, and practitioners Conduct competency assessments on BP measurement, cuff selection, and workflow adherence Provide quick-reference materials in all examination rooms (eg, visual aids and laminated guides) Provide regular and ongoing training in BP measurements	“So, I think the biggest thing that is office wide, one of the biggest barriers we find is that we’re not as well versed in doing BPs in younger children. So sometimes we have like complex cases where we need to and it was challenging... Are we even doing it the right way? Because we don’t do it that often... I think the biggest thing too is just like sizing and things like that. I just feel like sometimes we feel as though the cuff is the right size, but it’s probably, could have been like the size down or the size up. And if the reading’s normal, we’re just taking it but sometimes kids are in between sizes and we’re not sure exactly like which one we should be using or stuff.” HCP 19 “The main problem is that the MA does not follow the standard of care. That if electronic BP is abnormal with the right cuff, go to a manual with the right cuff. Or even come back after the visit finishes and retake the blood pressure when the patient calms down because many of these patients are anxious, you know about the visit because they are gonna have shot…you come to my office and look around what we have. You do an observation of all the medical assistants, taking blood pressure in different patients…you are going to notice behaviors…You are going to notice behaviors like documenting elevated BP and not doing a repeat BP…not doing a manual BP, and moving on because the doctor is in a rush…picking the wrong cuff.” HCP 15 “Honestly, at this point I don’t even know how well trained I am to check a BP because I don’t do it often and you know, I did receive training on doing a blood pressure, but my official training and doing blood pressure measurements was in medical school. So, I feel like I myself would want a refresher course.” HCP 9 “I would say training and again it’s a staffing issue, right? So, to train somebody perfectly to do these things perfectly and adhere to the guidelines, that takes time, that takes staff, that takes practice. And so I would say the other barrier would be training medical assistance because medical assistants come out of a 9-month program. It’s not very in-depth and so I think that they could use more training, more education about taking blood pressures and adhering to the guidelines. So, I would say training and lack of staff and lack of staff to do the training so it’s kind of like a snowball effect.” HCP 1
**Optimized EMR integration for hypertension detection (80%)**	
Enhance EPIC’s BP tracking system with automated percentile data and trend visualization Implement hard stops requiring BP documentation before EMR closure Improve alert sensitivity to reduce excessive flagging and prevent practitioner desensitization Automatically pull BP percentiles into the notes	“The computer threshold at certain ages is not the most accurate. So, I still like if I get an abnormal reading that I normally wouldn’t get on that patient, I usually again just like we’ll recheck it, talk to the doctor.” HCP 19 “I don’t know if the percentiles are flagging for over. I don’t know if it’s flagging for over the 90th percentile for age or the 95th percentile... I’m not sure, I think it flags over the 90th percentile maybe. Or if it’s below like the 50th percentile, I think if it’s a pressure under an absolute of 60. I don’t know what flags the low number, if it’s an absolute hypotensive number or if it’s related to the percentile. No, I mean I use the smart text to pull in prior blood pressures. Which I think is helpful instead of going back to other encounters and looking at the blood pressure on each individual visit, like you can pull prior blood pressures, which I find helpful. I think the flagging is helpful and I have a view where I can see that easily. Not everybody the same view/screen setup where the blood pressure measurements are obviously seen which may be a problem. I think when preset the residents, like when I’m looking at the resident charts, sometimes their view is not the same and they don’t see it. And I specifically asked like, “Are the measurements, okay? Is the blood pressure, okay?” And then they go back and look and they’re like “Oh, actually it’s high.” When it pulls into the exams in the notes, it’s not red, that might be helpful. So it’s red on the vital signs screen but when the measurements pull into the exam in the body of the note the percentiles go there but it’s not red. So, it’s not as obvious and that might be the only place where everybody’s in the note. So that might be helpful if it’s red in the note, when it links/pulls into the physical exam.” HCP 10 “I think there are some like improvements that could be made in Epic in terms of, it flags red, but I think it’s flagging any elevation and not stage 1 level. And so, like I think there’s some fatigue when it comes to Epic and people just see the exclamation point and then they just skip past it. Like there’s a window that says this should be repeated and I think we do follow that sometimes. But sometimes, when it’s busy, I think it’s easy to just click out of it and ignore it. I used to (complete problem list). I would say not very often…I’m sure there’s definitely times when I’ve had elevated readings that I did not add to the problem list.” HCP 17 “I think that could make a big difference. Like if (recommendations) popped up e.g., this (BP) is stage one, do this, this or like repeat if you haven’t repeated and this patient should be seen again for an office visit or give interval between office visits...and these were the last three blood pressure values on these dates, and this is the recommendation for follow up.” HCP 8 “Well, I think it could explain why it’s flagging it sometimes. So I think sometimes it flags it for a widened pulse pressure, sometimes it flat low sometimes cause the diastolic is too low. Sometimes there’s systolic is too high and it doesn’t necessarily let you know. I mean you can figure it out.” HCP 4 “The only thing that I think would be really helpful for us in the outpatient setting, it’s hard to get a good picture, is a flow sheet of what previous BPs have been and that would be a really helpful snapshot. Typically, what I do and maybe I just don’t know how to find it in epic, but if I want to see if this is an isolated high BP, I’ll go back through the notes and look at the vitals in previous notes.” HCP 3
Ensuring readily available and well-maintained BP equipment (44%)	
Standardize equipment availability across all clinics to ensure access to properly sized cuffs and machines Implement routine calibration protocols to maintain measurement accuracy Organize storage systems to facilitate quick and easy access to BP tools	“We have to make sure we have the right cuffs in the office for that age group, so it’s something that we are ordering now for our office. And with that age group now we have to use the Dynamap because it’s hard to do manual on a 4-month-old or a newborn.” HCP 21 “That’s what I go manually, because sometimes the machine is not calibrated enough and we are rushing due to lack of time, so we have to take a moment and say, ‘OK, let me do it with the right technique (calibrate it) the right way, so that I can get a right (accurate) number.’” HCP 13 “I will say - the cuff that I have here – the other day, I had to walk 12 examinations rooms to get the right cuff for one of my patients. My answer is that we need to figure it out a better way to have the equipment ready to be used. Sometimes the cuff is in a drawer, sometimes it’s in the cabinet, sometimes it’s nowhere. Also the cuff for the electronic machine is different from the cuff for the sphygmomanometer for a manual blood pressure. The MAs are in a rush and the bottom line is that they are going to take it with something that is close to them. If you come and you do an observation, you don’t say absolutely anything...they are using the wrong cuff... You know, sometimes the cuff is not fitting properly in the arm of the patient because the patient is too fat, but the patient is not that tall for the other cuff. That is another technicality that could be easily improve.” HCP 15 “I think that it should be universal... all our offices don’t function the same. And I don’t think that it is – I’ve worked in several other offices, and I don’t think it’s standard. I can come here, come into my office and do one thing but if I’m going to help another office, they might do something completely different. So, it all depends. Certain providers want certain things. I think that it should be standard across the board. I feel like every office should have the supplies that are needed to obtain a proper blood pressure. I know some offices don’t use the Dynamap machine at all, which is great, because the manual blood pressure is the best blood pressure. Then some offices only have the Dynamap machine. So, I think there should be a standard across the board. I think that would help a lot.” HCP 5
**Strengthening primary care physician–specialist communication and referral systems (36%)**	
Develop clear referral guidelines to ensure timely specialist consultation Enhance EPIC messaging to facilitate direct practitioner-to-specialist communication Use school-based clinics for BP monitoring and follow-up	“I think for the subspecialists like cardiology and nephrology, if they work to educate us with maybe like, I think, a clinical pathway. I’m a big fan of clinical pathways and I’ve done a lot of clinical pathway work. I think it’s probably the best way to educate and provide decision support and improve outcomes. So, I think that could be really helpful. And then I think other just like any other abnormal thing when you know like an allergist, for instance, if they do a blood pressure in their office and they notice that it’s high, I think communicating back with the primary care physician will help us see those patterns that we otherwise might chalk up to a nervous kid in the office.” HCP 3 “I wish they had somebody from cardiology or from nephrology, come and signed off on their skills because I feel like when they check their skills that the person always checking it may not be fully qualified. As I said we or I need a refresher course.” HCP 9 “I don’t know that they can help us with the detection in the office. I mean, if we get good guidelines and like a good clinical pathway, then we should... .” HCP 14 “I mean, I would if I had more training (on anti-hypertensives) or if they had seen like a Cardiologist once, and that’s what they recommended. Like I would be okay with refilling medications as long as they were not happening on any significant side effects.” HCP 17

Abbreviations: AAP, American Academy of Pediatrics; BP, blood pressure; EMR, electronic medical record; HCP, health care practitioner; MA, medical assistant.

### Intervention Characteristics

#### Barrier: Lack of Standardized Clinical Pathways

Clinicians consistently identified the absence of standardized, role-specific clinical pathways as a major barrier to consistent pediatric hypertension detection. Although guidelines exist,^1^ they are often perceived as overly complex or inaccessible in busy clinics. In the absence of embedded pathways, there is a substantial variation in practice for hypertension management, with clinicians relying on clinical judgment to determine how and when to perform BP remeasurement, documentation, and referral decisions. This variability leads to inconsistency among staff and across clinics.

#### Recommendation: Standardized Clinical Pathway for BP Measurement

Clinicians recommended the creation of simplified, visual clinical pathways that delineate next steps based on BP readings, role responsibilities and workflows, timing of remeasurement, and threshold for referrals. One clinician said, “I think it would be helpful to have a clinical pathway with what to do if there is a high blood pressure, as a standard guideline for providers…I don’t know if there’s like a specific smart phrase or dot phrase that would be utilized to be entered into the charts that would notify provider upon opening it that the patient had a high BP other than them reviewing the vital signs.” Integration of a clinical pathway into EMR via smart phrases and as a quick reference in examination rooms were emphasized to improve uptake.

### Characteristics of Individuals

#### Barrier: Inadequate Training and Confidence

Many clinicians expressed limited confidence in the accuracy of BP readings, particularly among newer medical assistants and nursing staff. Training inconsistencies were evident: some staff relied on informal peer instruction, whereas others had not received refresher training since initial orientation or even medical school for physicians. One clinician said, “Honestly, at this point I don’t even know how well trained I am to check a BP because I don’t do it often and you know, I did receive training on doing a blood pressure, but my official training and doing blood pressure measurements was in medical school. So, I feel like I myself would want a refresher course.” These gaps were particularly consequential for younger children, whose BP measurement requires size-specific cuff selection and behavioral adaptation strategies (eg, relieving anxiety and making sure cuffs are positioned properly).

#### Recommendation: Competency-Based Training and Educational Resources

Participants called for structured, role-specific, competency-based training programs focused on BP measurement technique, cuff selection, and recognition of red flags. They emphasized that training should be routine, ongoing throughout the year, accessible, and aligned with current American Academy of Pediatrics guidelines.

### Inner Setting

#### Barrier: EMR Design and Workflow Misalignment

EMR systems, although intended to support clinical workflows, were described as a barrier to hypertension detection. Clinicians noted that BP percentiles were often inconsistently displayed or not pulled into clinical notes. BP trend visualization tools were unavailable, and previous BP measurements from clinics were difficult to access, limiting clinicians’ ability to assess the chronicity of hypertension and develop thresholds for intervention. Clinicians also reported alert fatigue from inappropriate flagging of BP percentiles (eg, for a 1-point low diastolic BP) and noted the lack of hard stops that would require repeat measurements or follow-up documentation after an elevated BP is entered before EMR closure.

#### Recommendation: Optimize EMR Integration

Recommendations included redesigning EMR interfaces to (1) automate percentile calculations that are clinically relevant, (2) visually flag persistent elevations and display BP trends overtime, and (3) incorporate clinical decision support with actionable prompts based on BP thresholds. One clinician said, “I think that could make a big difference if [recommendations] popped up e.g., this BP is stage one, do this…or like repeat if you haven’t repeated and this patient should be seen again for an office visit or give interval between office visits...and these were the last three BP values on these dates, and this is the recommendation for follow up.” Clinicians also emphasized the need for standardized EMR views across roles and settings.

### Available Resources

#### Barrier: Lack of Equipment Availability and Maintenance

Measurement fidelity was compromised by inconsistent access to properly sized cuffs and calibrated BP devices. Clinicians frequently described searching multiple rooms for the correct equipment or settling for suboptimal alternatives due to time constraints. One clinician said, “I mentioned the supplies being an issue and making sure that all offices have the appropriate supplies for manual and Dynamap. And for those offices that do use Dynamaps quite often, making sure they can obtain a proper manual blood pressure cuff.” No standardized protocol existed for machine calibration or storage, and there was significant variability across clinic sites. In addition, some offices relied on oscillometric measurements, whereas others only performed manual BP.

#### Recommendation: Ensuring Readily Available and Well-Maintained BP Equipment

Clinicians recommended system-wide standardization of the type of equipment and its availability and maintenance, including dedicated storage systems, regular calibration schedules, and role clarity on equipment checks. These changes were seen as foundational to reliable BP measurement.

### Process

#### Barrier: Inefficient Communication and Referral Pathways

Coordination with subspecialty clinicians, such as cardiologists or nephrologists, was described as reactive and inconsistent. Primary care clinicians lacked clarity on referral thresholds and timelines, leading to overreferral in some cases and underrecognition in others. EMR-based messaging was underused or not standardized across practices.

#### Recommendation: Strengthening PCP-Specialist Communication and Referral Systems

Clinicians proposed the development of shared referral criteria codesigned with subspecialists as well as real-time EMR messaging templates to facilitate warm handoffs and clinical pathways. One physician said, “I think for the subspecialists like cardiology and nephrology, if they work to educate us with maybe like, I think, a clinical pathway.” In addition, they advocated for subspecialists to take a more active role in training, education, teaching, and facilitation of patient management decisions.

## Discussion

This study highlights the complexity of pediatric hypertension detection and provides a roadmap for developing clinician-informed system-level implementation strategies. Drawing from diverse clinicians across various clinical roles in primary care, we provide an in-depth understanding of the interconnected barriers that clinicians face, including unclear role expectations, lack of defined clinical pathways and workflows, limitations in EMR design, and fragmented referral processes. Guided by CFIR, this study expands previous work by identifying practical, clinician-informed practical strategies that align with the operational realities of pediatric care. Our findings offer a structured roadmap for designing implementation strategies grounded in both evidence and end-user experience.

Literature has extensively documented deficits in practitioner knowledge and lack of diagnostic consistency in pediatric hypertension.^9,10,12,14,16^ Bijlsma et al^14^ reported that most pediatricians measured BP only when risk factors were present and relied heavily on clinical intuition, often resulting in misclassification of BP readings. Similarly, Brady et al^23^ demonstrated that elevated BP were missed in nearly 90% of pediatric visits, particularly among less experienced practitioners or when potential cardiovascular disease risks were absent. These findings reinforce our study’s emphasis on the need for clinical decision support tools that automate BP percentile calculations and guide appropriate follow-up.^24,25^ Our study adds clinicians’ perspectives, highlighting that existing EMR systems often generate inaccurate or nonspecific alerts, lack intuitive visualizations of BP trends, and fail to support clinical decision-making.^26,27,28,29^ Clinicians identified these deficiencies as key contributors to diagnostic inconsistencies and missed opportunities for timely hypertension follow-up.

Although studies, such as Yoon et al’s^30^ work advocating for a paradigm shift in pediatric hypertension management by engaging PCPs, have largely emphasized specialist perspectives, few have explored how general pediatricians and their care teams actually navigate the complexities of hypertension recognition and referral.^9,14^ Our study fills this critical gap by capturing the voices of primary care practitioners, including general pediatricians, nurses, and medical assistants, who bear responsibility for accurate BP measurement, interpretation, documentation, and referral. Unlike previous literature, which has often overlooked the realities of primary care workflows, our findings illustrate that general pediatricians not only are eager for more support but also specifically call for increased specialist involvement in both education and the development of clear, accessible clinical pathways. These pathways would help delineate when and how to act on elevated readings and facilitate timely, appropriate referrals. This study demonstrates the need for specialists to play a more active role in the implementation of hypertension guidelines by developing partnerships with general clinicians to cocreate workflows that are realistic, role specific, and sustainable in busy practice settings.

Importantly, our findings also challenge the widely perceived notion that lack of time is the primary barrier to effective pediatric hypertension detection.^9,14^ Clinicians have previously acknowledged time constraints,^9,16^ but these challenges stemmed not only from the duration of patient visits but also from inefficient systems. Instead of only asking for more time, the clinicians in our study advocated for smarter workflows, clearer role-specific education, integrated clinical pathways, and user-friendly codesigned EMR systems. These preferences align with principles from implementation science, which emphasize that intervention usability and system design are as important as fidelity to clinical guidelines.^15,31,32,33^

Our study also demonstrates the importance of moving beyond traditional, one-time training efforts. Clinicians described the need for ongoing, role-specific education integrated into existing clinic routine and supported by accessible decision aids. This is consistent with earlier findings from Bello et al,^9^ who noted insufficient training on proper BP measurement among pediatric practitioners. Our study indicates that training alone is insufficient unless paired with role-specific training for various members of the clinical team and having systems-level support to embed ongoing continuous training focused on hypertension.

By applying the CFIR framework, we demonstrate how implementation barriers are interrelated.^15^ Measurement fidelity depends not only on access to appropriate equipment but also on adequate clinical pathways, staff training, and EMR features that support decision-making. This complements findings from Kroth et al,^34^ who observed that physicians face substantial burnout due to poor EMR design. Similarly, our study found that ineffective (hypersensitive or hyposensitive) alert systems and BP data buried in documents reduced the concern around high BP measurements, leading to missed follow-ups and poor hypertension detection. In line with alert fatigue literature,^24,25,35^ our participants emphasized the need for behaviorally informed EMR redesigns that prioritize clarity and relevance.

Referral systems and decision-making around appropriate referrals emerged as another area shaped more by system-level barriers than individual practitioner knowledge. Clinicians described a lack of shared referral pathways codesigned with subspecialists and inconsistent interclinician communication as obstacles that undermined consistent practice patterns, including when patients should follow up. This contrasts with earlier work that attributed overreferral and underreferral primarily to knowledge gaps.^30^ Our findings highlight the need for streamlined referral processes and coordinated care protocols, which can improve care coordination more effectively.

The diversity of health care delivery settings means solutions are unlikely to be one-size-fits-all. Private practices, large practitioner groups, and integrated health systems may need to adapt these findings to their own contexts, determining which strategies are broadly applicable (such as role-specific training or user-friendly decision aids) and that require tailoring to local resources and workflows (eg, referral systems and availability of subspecialty support). In addition, these insights also may help inform future hypertension guideline development by highlighting common implementation challenges and potential strategies to address them. Considering context using implementation science, understanding the diversity of health care systems, and incorporating these findings during guideline development could make recommendations clinically rigorous and more practical for clinical use, ultimately supporting improved recognition and management of pediatric hypertension.

### Limitations

This study has limitations. This qualitative study reflects the perspectives of health care clinicians within 1 regional pediatric health network, which may not capture the full range of experiences in other systems or geographic areas. Because interviews relied on clinician recall, responses may be influenced by memory limitations or a desire to present their practices favorably. Additionally, the study included only those clinicians who were willing to participate, and their beliefs and experiences may differ from those who chose not to engage.

## Conclusions

This study offers a detailed and actionable roadmap for improving pediatric hypertension detection by addressing implementation barriers at multiple levels. Rather than placing the burden of change solely on individual clinicians, our findings support a shift toward systems that enable consistent practice through thoughtful context-specific design and incorporation of end-user perspectives to focus on codevelopment of interdependent strategies, which include clinical pathways, ongoing training and education, redesign of infrastructure (eg, EMR and equipment), and better communication and care coordination. Future studies should move beyond identifying barriers and strategies to systematically evaluate their impact on implementation outcomes, such as feasibility, acceptability, adoption, and fidelity, and to test their long-term sustainability across diverse health care settings. Such work will be essential to ensure proposed solutions improve hypertension detection in the short term and become durable, scalable components of routine pediatric care.

## Notes

Supplement 1. **eTable 1.** Each CFIR domain and construct was defined and paired with questions to develop interview guides **eTable 2. **Characteristics of clinical sites of healthcare provider participants **eTable 3. **Sociodemographic characteristics of healthcare professionals **eTable 4. **Barriers to pediatric hypertension detection, aligned with CFIR domains and constructs, and illustrative excerpts from healthcare providers

Supplement 2.
Data Sharing Statement
