# Supplementary material for: Clinician Perceptions of Barriers and Strategies to Improve Pediatric Hypertension Detection
Source: JAMA Netw Open. 2026 Feb 27;9(2):e2560542. doi: 10.1001/jamanetworkopen.2025.60542 (PMC12949442; doi:10.1001/jamanetworkopen.2025.60542)
Supplement: Supplement 1. — eTable 1. Each CFIR domain and construct was defined and paired with questions to develop interview guides eTable 2. Characteristics of clinical sites of healthcare provider participants eTable 3. Sociodemographic characteristics of healthcare professionals eTable 4. Barriers to pediatric hypertension detection, aligned with CFIR domains and constructs, and illustrative excerpts from healthcare providers [file jamanetwopen-e2560542-s001.pdf]

Supplemental Online Content

Zaidi AH, Sood E, De Ferranti S, et al. Clinical perceptions of barriers and strategies to improve pediatric hypertension detection. *JAMA Netw Open*. 2026;9(2):e2560542. doi:10.1001/jamanetworkopen.2025.60542

eTable 1. Each CFIR domain and construct was defined and paired with questions to develop interview guides
eTable 2. Characteristics of clinical sites of healthcare provider participants
eTable 3. Sociodemographic characteristics of healthcare professionals
eTable 4. Barriers to pediatric hypertension detection, aligned with CFIR domains and constructs, and illustrative excerpts from healthcare providers

This supplemental material has been provided by the authors to give readers additional information about their work.

© 2026 Zaidi AH et al. *JAMA Network Open*. This is an open access article distributed under the terms of the CC-BY-NC-ND License, which does not permit alteration or commercial use, including those for text and data mining, AI training, and similar technologies.

eTable 1. Each CFIR domain and construct was defined and paired with questions to develop interview guides

CFIR Domain	Construct(s)	Definition	Sample Interview Questions
**Intervention Characteristics**	Evidence Strength & Quality; Complexity; Design Quality and Packaging	Perceived quality and clarity of the intervention, its usability, and perceived strength of supporting evidence.	Were you aware of these guidelines on how to measure BP and diagnose a patient with HTN?
			How do you determine if someone's BP is high or low?
**Characteristics of Individuals**	Knowledge & Beliefs about the Intervention; Self-Efficacy	Individual knowledge, attitudes, and confidence in performing tasks related to the intervention.	Do you think high BP is a problem for children? Why or why not?
			How confident do you feel about obtaining a patient's BP?
			Do you feel confident in dealing with elevated BP in your patients?
			Are there any knowledge gaps for you and your medical assistants and nurses?
**Inner Setting**	Implementation Climate; Readiness for Implementation	Organizational factors that influence implementation including workflow compatibility and internal resources.	What are the barriers in your practice to follow the guidelines when measuring or reporting a BP?
			What issues have you seen when recording BP in the EMR?
			How does your clinic infrastructure affect your ability to detect high BP?
**Available Resources**	Access to Knowledge & Information; Available Resources	Tangible resources including time, staff, space, and equipment necessary to support implementation.	Do you have enough clinical space to carry out the clinical activities?
			What equipment do you have for measuring BP?
**Process**	Engaging; Executing; Reflecting & Evaluating	Steps and engagement strategies used to implement and sustain an intervention.	How could sub-specialists help you detect high BP or HTN?
			If you had to change the guidelines, what would you change to make detecting HTN easier?

CFIR, Consolidated Framework for Implementation Research; HTN, hypertension; BP, blood pressure; EMR, electronic medical record

© 2026 Zaidi AH et al. *JAMA Network Open*. This is an open access article distributed under the terms of the CC-BY-NC-ND License, which does not permit alteration or commercial use, including those for text and data mining, AI training, and similar technologies.

eTable 2. Characteristics of clinical sites of healthcare provider participants

Site	Number of providers	Child Opportunity Index	Gap in care >1 year	Roles
1	4	Very High	4.2%	2 MD, 1 Nurse Manager, 1 Nurse
2	2	Very High	0.9%	1 MD, 1 Advanced Practice Provider
3	3	Low	7.4%	1 MD, 1 Nurse Manager, 1 Medical Assistant
4	2	Low	5.6%	1 MD, 1 Nurse Manager
5	2	Moderate/low	8.8%	1 MD, 1 Nurse
6	2	Moderate/low	3.7%	1 MD, 1 Medical Assistant
7	3	Low	5.6%	1 MD, 1 Nurse Manager, 1 Medical Assistant
8	2	Low	3.9%	1 MD, 1 Nurse Manager
9	2	Low	28%	1 MD, 1 Nurse
10	3	Low	5.1%	1 MD, 1 Nurse Manager, 1 Nurse

© 2026 Zaidi AH et al. *JAMA Network Open*. This is an open access article distributed under the terms of the CC-BY-NC-ND License, which does not permit alteration or commercial use, including those for text and data mining, AI training, and similar technologies.

eTable 3. Sociodemographic characteristics of healthcare professionals

Healthcare Professionals (N=25)	N (%)
**Age (years)**	
25-35	4 (16%)
35-44	9 (36%)
45-54	5 (20%)
55-64	7 (28%)
**Gender (Female)**	22 (88%)
**Role**	
Physician-in-charge/regional chief	6 (24%)
Primary care physician	5 (20%)
Nurse manager	6 (24%)
Medical assistant	3 (12%)
Nurse	4 (16%)
Advanced practice nurse	1 (4%)
**Educational Background**	
Medical degree (MD, DO)	11 (44%)
Bachelor's degree (nursing, psychology, behavioral science)	7 (28%)
Master's degree (nursing, psychology)	4 (16%)
Associate's degree	3 (12%)
**Years of Service at NCH**	
<5	5 (20%)
5-10	10 (40%)
11-15	3 (12%)
15-20	3 (12%)
>20	4 (16%)
**Clinic Location's COI**	
Very low/Low	15 (60%)
Moderate	4 (16%)
High/Very high	6 (24%)

© 2026 Zaidi AH et al. *JAMA Network Open*. This is an open access article distributed under the terms of the CC-BY-NC-ND License, which does not permit alteration or commercial use, including those for text and data mining, AI training, and similar technologies.

© 2026 Zaidi AH et al. *JAMA Network Open*. This is an open access article distributed under the terms of the CC-BY-NC-ND License, which does not permit alteration or commercial use, including those for text and data mining, AI training, and similar technologies.

eTable 4. Barriers to pediatric hypertension detection, aligned with CFIR domains and constructs, and illustrative excerpts from healthcare providers

CFIR domain and associated constructs	Major Themes	Healthcare Provider Excerpts

© 2026 Zaidi AH et al. *JAMA Network Open*. This is an open access article distributed under the terms of the CC-BY-NC-ND License, which does not permit alteration or commercial use, including those for text and data mining, AI training, and similar technologies.

**Intervention Characteristics: Design Quality and Packaging**<br><br>Refers to how well an intervention (like BP guidelines) is designed, presented, and packaged for clinical use. If materials are confusing, incomplete, or not user-friendly, implementation is challenging.	**Lack of Standardization in HTN Guidelines**<br><br>• Providers struggle with adherence due to lack of awareness and perceived complexity.<br>• Guidelines are inconsistently applied across clinics, leading to variability in care.<br>• Limited awareness of post-BP follow-up recommendations result in inconsistent patient management.	"We don't always average (BP readings) because sometimes the first one was clearly like spurious or like so out of range compared to the next one. I think we tend to pick the best blood pressure that we have instead of averaging. We don't do three separate visits. We should, but we don't. And I think part of that is time and again, sort of our suspicion of whether we think it's white coat hypertension or real hypertension". **HCP 17**<br><br>"I think they do (multiple BP readings) most of the time. I think there are definitely instances when we are pressed for time and maybe they're not doing that second manual blood pressure. I know the providers have brought that up before". **HCP 12**<br><br>"I was not aware of that (about taking a second BP measurement for high BP and also averaging them). I guess never did that because I am just hearing about it". **HCP 24**<br><br>"Some (steps of the guideline), what you said, we do that – but the position…it depends on what the age group is… but we do just manual readings…So we do the manual and repeat again". **HCP 21**<br><br>"Yeah, (guideline use) that's variable. We go by the definition of AAP, but it's one type / episode of high BP (HTN) cannot be diagnosed for kids. So, it depends on the situation. Sometimes we bring them to repeat, if they are nearby, just for measurements. Maybe another time in a week and the doctor not seeing them, just getting the blood pressure. Or, I have even done situations when I have seen that a person has persistently been coming here for multiple follow ups…So, then we tell the school nurse to measure it, make a chart and send it to us for seven days". **HCP 22**

© 2026 Zaidi AH et al. *JAMA Network Open*. This is an open access article distributed under the terms of the CC-BY-NC-ND License, which does not permit alteration or commercial use, including those for text and data mining, AI training, and similar technologies.

**Characteristics of Individuals: Knowledge and Beliefs**<br><br>Captures the training, knowledge, and confidence of the people expected to use the intervention (e.g., pediatricians, nurses, MA). If they are unaware of the guidelines, or do not believe they can apply them, implementation becomes difficult.	**Limited Training and Knowledge Gaps**<br><br>• Providers lack adequate training on BP measurement, particularly in younger children.<br>• Uncertainty in guideline application (e.g., when to repeat BP readings, manual versus automated BP methods).<br>• Some providers lack confidence in initiating HTN treatment.<br>• Limited access to educational resources for both providers and caregivers.	"(Limited comfort for prescribing anti-hypertensives)…I cannot give you a specific reason. I think it's not something we do every day. I feel it's necessary. But I feel as a pediatrician, I need more education on that". **HCP 22**<br><br>"I would never feel comfortable to start a medication to treat hypertension. I feel fine continuing them, managing them, when patients come in on them. Lots of NICU babies come in on hypertension medications, cardiac kids come in on anti-hypertensive medications. And I feel sort of monitoring hypertension, but I would never make an adjustment of their medication without talking to the Cardiologist or Nephrologist first. And I would never start a medication without consulting". **HCP 20**<br><br>"The only challenges we've faced is with newer staff like new nurses that were orienting. A lot of times they're not because we do manual. Sometimes they're not as confident in their manual blood pressures, so it takes a little bit more time to train and orient and recheck a few more blood pressures to make sure that they're accurate. I know sometimes there's confusion about having the correct supply with the right size of blood pressure cuff. And then also in the event that we do use a Dynamap, I know we have two different versions of Dynamaps in our office, so making sure we have the right cuff size for all the different varieties of dynamaps that we have. The plugs can vary on the blood pressure cuff so sometimes that can cause a delay". **HCP 11**

© 2026 Zaidi AH et al. *JAMA Network Open*. This is an open access article distributed under the terms of the CC-BY-NC-ND License, which does not permit alteration or commercial use, including those for text and data mining, AI training, and similar technologies.

**Inner Setting: Compatibility & Available Resources (EMR Challenges)**<br><br>Looks at whether the intervention "fits" with the organization's existing systems like the EMR. If the EMR doesn't flag abnormal BPs clearly or creates alert fatigue, it blocks effective implementation.	**Inefficiencies in EMR for BP Management**<br><br>• EPIC (health record software) inconsistently flags BP readings (missed, persistent, or unclear thresholds).<br>• No easy way to track BP trends over multiple visits.<br>• Alert fatigue leads to providers ignoring or skipping notifications.<br>• No "hard stops" preventing chart closure before addressing BP abnormalities.	"I rely on the flag, that will then turn the blood pressure measurement red with an exclamation mark as an abnormal blood pressure. The percentiles are there to tell me if the blood pressure is out of range, too high or too low. So, the flag, the exclamation mark, just alerts me and the percentiles are there". **HCP 10**<br><br>"I feel like we don't typically always see percentiles from EPIC that get pulled into the patient's progress note. So, I don't know if I can really respond to that besides getting that notification that pops up in epics saying that this was an abnormal BP". **HCP 11**<br><br>"I know it is kind of cumbersome when I have to go into vitals. Then I have to hit the little because I don't put the vitals in all the time, so it's like cumbersome for me to have to do it. So there might be a, maybe a different way. I don't know. I wonder if they could. Like if it flags an abnormal blood pressure, if there could be like a pop up that like you can put the second blood pressure in rather than having to go through the tabs. You know, they're going to vitals and then you have to hit the little plus sign... so I don't know, maybe there's a way they can streamline that a little differently". **HCP 14**<br><br>"What I like to learn in EMR if it's possible is like how to place sets of orders. So yeah, if somebody is overweight, you know this is the set of labs that I order, some people have learned that. And instead of ordering the lab separately and then sometimes forgetting what to order. So, for example, cardiology would say, well, if somebody you know before you send us this patient for elevated blood pressures, we want you to do this set of lab work. And so, this way I just could click on you know elevated blood pressures or whatever instead of yeah, because I might order metabolic panel and forget to order the lipid panel or something of that type. So, that would be the problem like that I would like to have like sets of specific labs for specific conditions". **HCP 9**

© 2026 Zaidi AH et al. *JAMA Network Open*. This is an open access article distributed under the terms of the CC-BY-NC-ND License, which does not permit alteration or commercial use, including those for text and data mining, AI training, and similar technologies.

**Inner Setting: Compatibility and available resources**<br><br>Looks at whether the intervention "fits" with the organization's existing systems like the EMR. If the EMR doesn't flag abnormal BPs clearly or creates alert fatigue, it blocks effective implementation.	**Inadequate BP Equipment and Accessibility Issues**<br><br>• Limited access to properly sized BP cuffs, especially for infants and adolescents.<br>• Uncalibrated or malfunctioning BP machines contribute to measurement errors.<br>• BP machines and cuffs are not always stored in accessible locations, causing delays in patient care.	"Doing a manual BP is trickier, it's a harder technique, to do it. I have to go find the cuff, they're not in the exam rooms, so that's always an added few minutes. And the dynamap are right in the hallway, right outside the rooms, it's easier for me to do it myself with the automated machine. And I'll feel good about that being accurate based on my technique and the way to patient is situated". **HCP 10**<br><br>"Yeah, I mentioned the supplies being an issue and making sure that all offices have the appropriate supplies for manual and Dynamap. And for those offices that do use Dynamaps quite often, making sure they can obtain a proper manual blood pressure cuff because you know that should be our second or third step, if you're doing a repeat blood pressure, OK, the Dynamap has been inaccurate. Maybe the first two readings. So let's just check it manually…I have people that come to primary care from hospitals often and even other offices, and they're so used to taking an electronic blood pressure that I hand them a manual blood pressure and they're like oh, haven't done this in a long time. So that's not always great either". **HCP 11**<br><br>"Our roomers are all new. Almost all new. And we've had some turnover. And we are sort of perpetually short of support staff. Seemingly, we just get up to speed and then something happens and we're behind it again. So they're always moving. I mean, the pace of primary care moves pretty fast and the access of the Dynamap can sometimes have to wait for it". **HCP 8**

© 2026 Zaidi AH et al. *JAMA Network Open*. This is an open access article distributed under the terms of the CC-BY-NC-ND License, which does not permit alteration or commercial use, including those for text and data mining, AI training, and similar technologies.

**Process: Engaging (Coordination with Subspecialists)**<br><br>Examines how well key people outside the immediate team (like sub-specialists) are involved. Poor coordination creates gaps in referral and follow up.	**Limited PCP-Specialist Coordination in HTN Care**<br><br>• Lack of clear referral criteria for cardiology and nephrology.<br>• PCP have limited direct communication with specialists, delaying decision-making.<br>• School-based health centers are underutilized for BP re-checks.	"I don't know. I think personally I would like to know (sub-speciality education). I mean, I guess it goes with detection, but it's more so because sometimes I felt like we send folks to Cardiology for like stage one that hasn't improved with dietary modifications. And then I feel like it's not - they're just like," keep going with diet and exercise" and like nothing really changes either. So, I guess then it's sort of like decreases motivation to detect it. So, I guess yeah, maybe just real advice on like when they would treat. If they have any pearls on like ways to sort of tease out if its white coat hypertension or not. And if in their experience like missing or sort of like ignoring certain levels of elevated blood pressure really led to serious consequences, I think might kind of emphasize the importance of it more to us". **HCP 17**<br><br>"So we don't have a set criteria for that. I think we refer at the earliest whenever we feel that "Okay this is a persistently, consistently high blood pressure coming in multiple episodes". Or patient has any other correlated symptoms or comorbidities like obesity, has high lipids in the lipid panel or we have headache or any other palpitations or any other symptoms we just send them". **HCP 22**<br><br>"I do. But as I mentioned, the flow for us and Nemours has been that the cardiologists manage this and they are going to start the mitigation and they are going to follow the patients. OK, there is a tendency at this moment that everybody is overwhelmed because of the shortage of things that we have. And there is a tendency that now we are looking into it instead of specialist. OK. And send more things to do like sending a hypothyroidism to be managed by the primary care doctor, diabetes to be managed by the primary care doctor or complicated asthma to be managed by the primary care doctor instead of the pulmonologist and see 20 patients a day. OK. And also manage a psychiatric medication for the patients with depression. There is a mismatch. It is not going to be good care". **HCP 15**<br><br>"I think we're working parallel. We are the frontliners, catching the patient, like in a screening process and then the patient goes to them, and they manage the patient. And pretty much we try to be sure that the patient follows through with the specialist when we see them in the annual checkup. But to be honest, there is

© 2026 Zaidi AH et al. *JAMA Network Open*. This is an open access article distributed under the terms of the CC-BY-NC-ND License, which does not permit alteration or commercial use, including those for text and data mining, AI training, and similar technologies.

		not much interaction. When you are around a long enough to know people I can contact them by e-mail, by messaging in epic, OK. But also, the longer you work in the satellite clinic, then you don't know anybody, you just rely in the visit with the expert rather than, I will say a real co-management of the patient. OK. Where we are really. Multidisciplinary team for the best care of the patients…I think we are isolated...we have not been really interacting much". **HCP 15**

CFIR; consolidated framework for implementation research, BP, blood pressure; HCP, healthcare provider; AAP, American Academy of Pediatrics; MA, medical assistant; EMR, electronic medical record; HTN, hypertension, dynamap; oscillometric blood pressure device, NICU; neonatal intensive care unit.

© 2026 Zaidi AH et al. *JAMA Network Open*. This is an open access article distributed under the terms of the CC-BY-NC-ND License, which does not permit alteration or commercial use, including those for text and data mining, AI training, and similar technologies.
