# Supplementary material for: Clinician Perceptions of Barriers and Strategies to Improve Pediatric Hypertension Detection
Source: JAMA Netw Open. 2026 Feb 27;9(2):e2560542. doi: 10.1001/jamanetworkopen.2025.60542 (PMC12949442; doi:10.1001/jamanetworkopen.2025.60542)
Supplement: Supplement 2. — Data Sharing Statement [file jamanetwopen-e2560542-s002.pdf]

Data Sharing Statement

Zaidi. Clinician Perceptions of Barriers and Strategies to Improve Pediatric Hypertension Detection. *JAMA Netw Open*. Published February 24, 2026.
doi:10.1001/jamanetworkopen.2025.60542

Data
Data available: Yes
Data types: Deidentified participant data
How to access data: Request can be submitted through the research coordinators at the email: varsha.zadokar@nemours.org
When available: With publication

Supporting Documents
Document types: Statistical/analytic code, Informed consent form
How to access documents: Request can be submitted through the research coordinators at the email: varsha.zadokar@nemours.org
When available: With publication

Additional Information
Who can access the data: Anyone requesting the data
Types of analyses: Any purpose
Mechanisms of data availability: After approval of proposal with a signed data user agreement
